# Differences in Portion Sizes in Brazil, France, and the USA

**DOI:** 10.3390/foods13030455

**Published:** 2024-02-01

**Authors:** Matthew B. Ruby, Marle S. Alvarenga, Paul Rozin

**Affiliations:** 1 Department of Psychology, Counselling and Therapy, School of Psychology and Public Health, La Trobe University, Wodonga, VIC 3690, Australia; 2 Program of Post Graduation in Public Health Nutrition, School of Public Health, University of São Paulo, São Paulo 01246-904, SP, Brazil; marlealv@usp.br; 3 Department of Psychology, University of Pennsylvania, Philadelphia, PA 19104, USA; rozin@psych.upenn.edu

**Keywords:** portion sizes, culture, food, attitudes, gender

## Abstract

Portion size is recognized as a major determinant of food intake, at least over the short term, and could be related to overconsumption and obesity. In this study, we developed and evaluated a new visual measure of portion size (PS), examined whether the PS of chicken, ice cream, and soda varied among people in Brazil, France, and the USA, and tested whether PS was related to gender, body mass index, body weight, and socioeconomic status. We conducted a cross-sectional study using online convenience samples of university students (total *N* = 1391). Across all three foods, French personal and country PSs were significantly smaller than the other three countries. Estimated country PS was reliably larger than personal PS. Women’s personal PSs were smaller than men’s, but women’s and men’s estimates for country PS were similar. French personal and country PSs were the lowest. Some PSs had a weak but significant correlation with SES but were not significantly related to either weight or BMI. The study confirms French-American differences in personal PS and demonstrates that perceived norms correspond to individual PS.

## 1. Introduction

The link between portion size and food intake is well-established and has become a major area of inquiry. Over the short term, the increased consumption of larger food portions is well established, and there is some evidence for long-term effects [1,2,3,4,5]. Indeed, the World Health Organization Technical Staff [6] affirmed that establishing healthy eating patterns in early development, which include appropriate portion sizes of nutritious, non-energy-dense foods, may be an important factor in obesity prevention. Portion size is of particular interest because it is easier to control than many other determinants of food intake. It is important to highlight that we are using the term portion size to refer to the amount of food offered and chosen by people since the term serving size—the amount listed on the nutrition information label of foods or the commercially prepared packaging and in dietary guidelines—is often confounded with it [7,8].

Assuming a food is reasonably palatable, availability, partially accounted for by portion size, is a major determinant of intake at the level of the meal [1,9,10,11,12]. A portion size effect (larger portions producing higher intake) has been observed across a variety of food types, environmental conditions, and study populations [1,3,9,13,14,15] through both directly manipulating portion size in laboratory studies and self-report or observation studies. Furthermore, this has been shown to vary between countries—a substantially smaller portion size in France as opposed to the USA has been observed in restaurants and food stores [14]. This is one potential explanation for the obesity rate in France, which is almost half of the rate in the USA [16]. Across many studies, women tend to estimate portion sizes more accurately than men or report smaller portion sizes [17,18,19,20,21,22]. Some studies have shown that people with a higher body mass index (BMI) tend to report larger portion sizes [21,23,24,25]. Associations with socioeconomic status (SES) are less clear—although some studies have found that people of lower SES report larger portion sizes [26,27], others find no significant differences regarding SES [28,29]. 

The great majority of portion-size research has been performed in the Anglo-developed world, but there has been some exploration in non-Anglo-European [6,14,23,30,31,32,33] and Asian [27,34,35] countries. There has also been some recent portion-size work performed in non-Western developing countries such as Brazil. For example, experiments by Japur and Diez-Garcia [36] showed that participants tended to overestimate the portion size of high-energy foods (e.g., margarine and sugar) and underestimate the portion size of low-energy foods (e.g., cauliflower and papaya). Furthermore, 24 h dietary recall data from Pereira et al. [37] indicated that larger portions of some foods (e.g., pizza, meat, and soft drinks) were associated with being overweight. 

In three thorough reviews of the literature on portion size [1,11,30], researchers have highlighted the importance of the environmental context (including the presence of other eaters) and identified some gaps in the current literature. There are good data suggesting that portion size affects the amount eaten in the real world over a few meals or days (there are some data suggesting the long-term enhancement of food intake over the week [11,38,39]). However, two laboratory studies that imposed larger portion sizes on subjects for more than a week found minimal compensation in subsequent meals [38,39]. In a field experiment, very large portion sizes (1600 calories) every weekday for six months led to substantial weight gain [40]. Large portion sizes of energy-dense foods have been considered a central feature of the dietary environment related to the rise in obesity since the 1990s [5,41]. In the USA, portion sizes for commonly available foods have increased during the past 30–40 years, coinciding with the timeframe in which the prevalence of obesity has increased [42,43]. 

People tend to consume palatable food that is in front of them (for amnesiacs, even minutes after they have completed a full meal) [10]. For normal eaters, portion sizes suggest cultural consumption norms. People do not usually consume two appetizers, main courses, or desserts at a meal– rather, they typically finish one of each course. This has been called unit bias, measured as a substantial increase in snack consumption when the “unit size” is larger (e.g., M&Ms self-served with a teaspoon or tablespoon), even though abundant additional food is available [44]. Similarly, Geier, Rozin, and Doros [44] showed that people rate two different platters of the same food, one with each food 50% larger, as approximately the same number of calories. Another account of the portion size effect is “value-priced sizing”, which links portion size with cost [42]. The price is cited by shoppers as a major influence on product choice, making foods and drinks in larger portions and packages more appealing because they often cost less in relative terms [1,3,11,45,46]—see Steenhuis [2] for a list of other proposed mechanisms.

In this context, the objectives of the present study were to (1) examine whether portion sizes for three specific foods (chicken, soda, and ice cream) vary between people sampled from Brazil, France, and the USA; (2) examine the relationship between perceived country portion size norms and individually selected portion sizes; and (3) explore the relationship between portion size and participant gender, body mass index, body weight, and socioeconomic status. 

The study extends prior work on portion size in three ways: (1) A new self-report technique is used, in which people report their usual portion size by selecting from a standardized visual array, using familiar objects as anchors. For chicken, we used a deck of cards; for ice cream, a tennis ball; and for soda, a sixteen-ounce glass with eight different levels marked, held by a visible person so that the size of the glass is clear. (2) Comparisons were performed across three countries, for one of which (Brazil) there is currently very little data [47]. (3) We measured not only personal portion sizes but also perceptions of country-level norms.

## 2. Materials and Methods

### 2.1. Participants and Design

From late 2010 to 2012, we recruited a total of 1391 participants from universities in three countries: 583 participants were students from the University of São Paulo in Brazil (62% women, *M_age_* = 21.3, *SD_age_* = 2.46) and were informed of the study via an email sent through the university’s academic listservs; 441 participants were students from the Université de Nantes in France (62% women, *M_age_* = 21.6, *SD_age_* = 1.46) and were informed of the study via an email sent to all students of the Audencia Nantes School of Management; and 367 participants were students from the University of Pennsylvania (UPenn) in the USA (65% women, *M_age_* = 21.5, *SD_age_* = 3.21) and were informed of the study via announcements in introductory and social psychology courses, and via an email to all graduate students at the university. The questionnaire was hosted on Surveymonkey.com and was described as “a survey on body image and attitudes toward food and physical activity” and was part of a larger project that also measured people’s attitudes toward meat eaters and vegetarians (previously reported in [47]). We aimed to collect at least 300 participants per country, which would exceed the sample size needed to detect a small-to-medium effect of *d* = 0.25 via an independent sample *t*-test at 80% power (required *n* = 258).

All participants completed the survey on a voluntary basis. Following local norms, undergraduate students at UPenn received course credit for their participation, and graduate students at UPenn were entered into a draw for a $100 cash prize. The US sample was intentionally collected from both graduates and undergraduates for two reasons: (1) In the US, many undergraduates live in dormitories, and we wanted a good representation of US students living off campus, as is the case in other countries. (2) US undergraduates are somewhat younger than undergraduates from other countries. For a more detailed description of sample demographics, see Table 1.

To guard against careless responses and to ensure more representative cross-cultural comparisons, we systematically excluded data from any participants who had either left more than 30% of the questionnaire blank (Brazil: 95, France: 67, and USA: 13), were outside the age range of 18–30 or did not specify their age (Brazil: 50, France: 6, and USA: 43), did not specify their sex (Brazil: 1, France: 0, and USA: 9), or were born outside of their university’s country or had lived the majority of their life since age 10 outside of said country (Brazil: 2, France: 18, and USA: 64). 

All participants provided informed consent before beginning the online survey.

### 2.2. Materials

An initial questionnaire was developed in English and pilot-tested with university students in the USA. The questionnaire was then translated into Brazilian, Portuguese, and French by native speakers of the relevant languages. A back translation into English was performed by a different set of bilingual translators, and discrepancies were resolved via discussion between the translators. 

At the end of the survey, participants provided a range of demographic data (e.g., age, gender, ethnicity/race (except for France, where this is not permitted), self-reported height and weight (to calculate body mass index, BMI), and socioeconomic status (SES; 1 = lower, 2 = lower middle, 3 = middle, 4 = upper middle, and 5 = upper). Other measures not included in this report explored body image, attitudes toward beef and vegetarians (published separately; 21), and general attitudes toward food, eating, and exercise. 

### 2.3. Food Portion Size Questions

We employed a new self-report technique, in which people selected their usual portion from a visually presented array of different portion sizes, which were anchored by a standard that was highly similar across countries. By using this international standard, we aimed to reduce bias in portion size recall. For chicken, we used a deck of cards (approximately 2.5 × 3.5 × 0.5 in); for ice cream, a tennis ball (approximately 2.6 in diameter); and for soda, a sixteen-ounce cup with eight different levels marked, held by a visible person, so that the size of the cup was clear. Participants also indicated what they considered to be country norms (average or most common portion size) using the same array.

**Figure 1:**
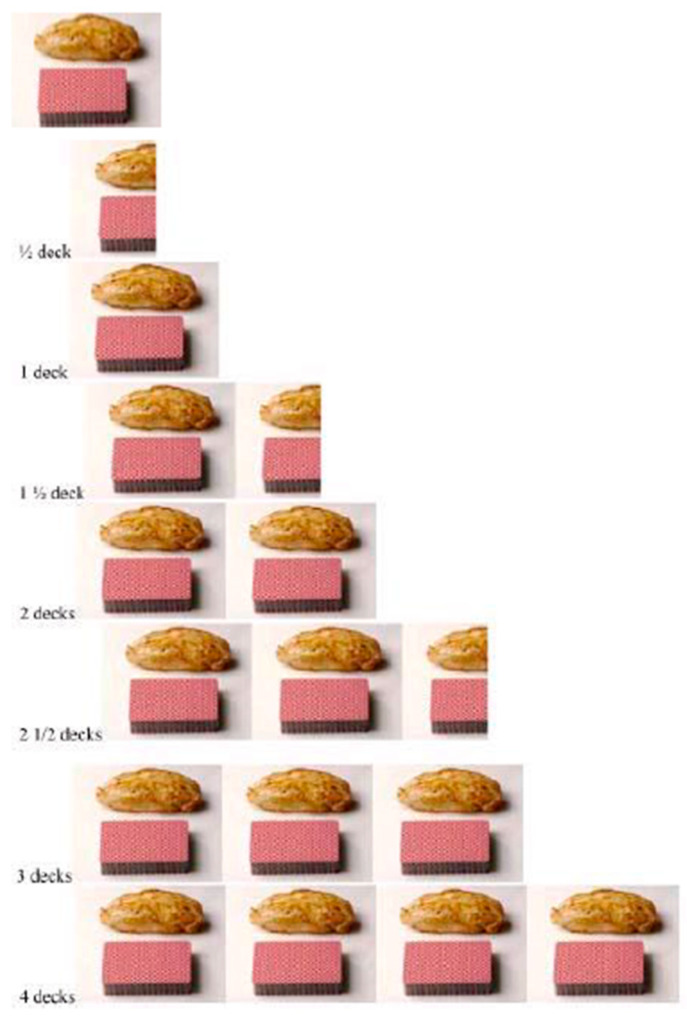
Choice of chicken portion sizes.

The chicken portion size was assessed using the following items: (1) “Using the deck of cards as a reference, please choose the amount of chicken which corresponds to what you would normally serve yourself for a meal (or you can choose 0 if you do not eat chicken)” and (2) “Using the deck of cards as a reference, please choose the amount of chicken which corresponds to the size that is most commonly consumed by adults in (your country)”. (See Figure 1).

**Figure 2:**
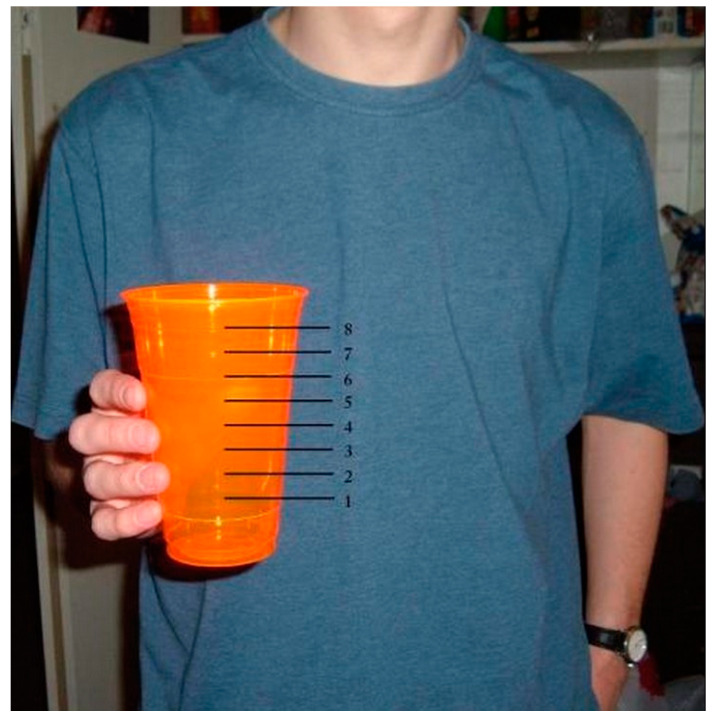
Choice of soda portion sizes.

Similarly, soda portion size was assessed with the following items: (1) “Imagine that you are going to pour yourself an amount of regular coke (without ice) that you would normally drink into a 16 oz (473 mL) cup (without ice but at the temperature you prefer). Please choose the number which corresponds to how much coke you would most likely serve for yourself when thirsty. If you do not like coke, please imagine you are pouring out another similar beverage of your choice. If not soda, it should be a beverage that contains a similar amount of sugar as standard soda beverages, such as apple juice” and (2) “Now please choose the number which corresponds to how much regular coke the average (Brazilian, French, or American person) would serve for himself/herself in one sitting” (See Figure 2).

**Figure 3:**
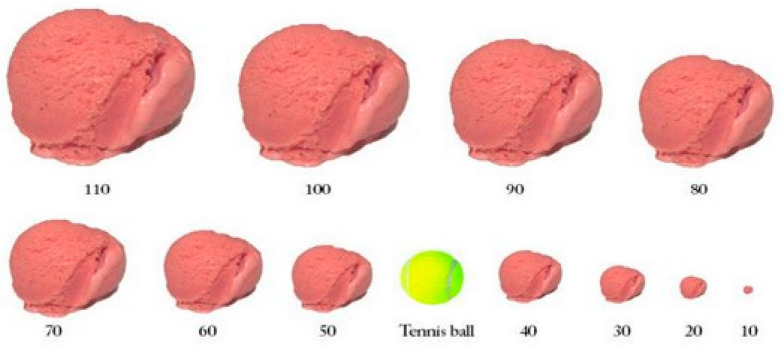
Choice of ice cream portion sizes.

Ice cream portion size was assessed with the following items: (1) “Choose the scoop of ice cream (any number from 10 to 110) that is closest to what you would normally serve yourself” and (2) “Choose the scoop of ice cream (any number from 10 to 110) that corresponds to the size that is most commonly eaten by adults in [Brazil, France, or the USA]”. (See Figure 3).

### 2.4. Statistical Analyses

Analyses were conducted using SPSS 25.0 (Statistical Package for Social Science Inc., Chicago, IL, USA) software. Given the large sample size, the level of significance was set at *p* < 0.01, 2-tailed. 

For each of the three foods, we conducted a series of 2 (gender) X 3 (country) ANOVAs. We did this separately for personal portion size, country portion size, and the difference between personal and country portion size. Pairwise comparisons used Scheffe’s test. Eleven participants (0.6% of the sample) indicated that they did not eat chicken, and as such, they were not included in the analysis of chicken personal portion size.

A copy of the anonymized dataset is available on the OSF (https://osf.io/umer3/?view_only=8280bfd1bfc142de95a967a6326ab779, accessed on 1 December 2023).

## 3. Results

Table 2 presents the mean personal and country portion size for each of the 18 groups (three foods by two genders by three countries). In presenting the results in tables and graphs, the data from ice cream were divided by 10 so that all the means fell in the same range. 

### 3.1. Personal Portion Sizes

For all three foods, there was a significant effect of gender (all *p* < 0.001), such that men’s portion sizes were larger than women’s. Men’s mean portion sizes (equally weighting countries) were 49% higher for chicken, 22% higher for soda, and 13% higher for ice cream. 

For all three foods, there was also a significant effect of country (all *p* < 0.001). For chicken, the mean portion size was significantly larger in Brazil and the USA than in France. For soda, the mean portion size was significantly larger in Brazil than in France and the USA. For ice cream, the mean portion size was significantly larger in Brazil than in the USA, which was significantly larger than in France. France had the smallest portion size across all three foods. Combining portion sizes, equally weighted by gender and food, and using the French portion size as the base, Brazilian sizes were 21% larger, and American sizes were 13% larger. 

None of the gender-by-country interactions were significant. For a summary of all *F* statistics, see Table 3.

### 3.2. Country Portion Size

There was a significant effect of gender for soda (*p* < 0.001), such that men’s estimates of the average portion size in their country were 6% smaller than women’s. There was also a significant effect of gender for chicken (*p* < 0.001), such that men’s estimates were 5% larger than women’s. The absence of a strong gender effect is to be expected since all respondents were asked to estimate the most common portion size for their whole country.

For all three foods, there was also a significant effect of country (all *p* < 0.001). For chicken and ice cream, all countries significantly differed from one another, with the largest portion in the USA, followed by Brazil, then France. For soda, the mean portion size was significantly smaller in France than in the other two countries. For all three foods, the French portion size norm was significantly lower than for both other countries.

Combining portion sizes, equally weighted by gender and food, and using the French estimated country portion size as the base, American sizes were 42% larger and Brazilian 32% larger.

The interaction of gender with country was significant for chicken (*p* = 0.004). An examination of simple effects revealed that men’s estimates were significantly larger than women’s in the USA (*p* < 0.001) but not in Brazil (*p* = 0.81) or France (*p* = 0.04).

### 3.3. Country Versus Personal Estimates

For all three foods, there was a significant effect of gender (all *p* < 0.001), with women reporting a significantly larger difference between their personal portion size and their estimated country portion size. Men’s mean differences in portion sizes (equally weighting countries) were 476% higher for chicken, 310% higher for soda, and 287% higher for ice cream. 

For all three foods, there was also a significant effect of country (all *p* < 0.001). For chicken and soda, the mean portion difference was significantly larger in the USA, followed by Brazil, and then France. For ice cream, the difference was significantly larger in the USA than in Brazil and France. 

None of the gender-by-country interactions were significant.

### 3.4. Other Notable Findings

One might expect that individuals who consumed larger portions of one food would do so for another, and this would manifest in a positive correlation between the personal portions selected across the three foods. We calculated these correlations separately for men and women, with the results displayed in Table 4. For each gender, we calculated three correlations to cover the three pairings made from three foods, and we averaged these. All were significant and positive but rather low (men’s mean *r* = 0.20 and women’s mean *r* = 0.12). The consistency of country portion size estimates across foods was much more substantial, with both mean *r* = 0.36.

One might also expect portion size to be larger for people with higher BMIs, but there were no significant correlations between BMI and personal portion size for any of the foods for either gender (Table 4). Similarly, there was no significant correlation between country portion size and BMI for any of the foods for either gender. One could also predict that body weight, more so than BMI, should correlate with portion size (i.e., the higher the weight, the more energy needed, and the greater the intake), but again, there was no significant correlation for any of the foods for either gender. Among men, the SES was negatively correlated with half of the portion measures (soda country, ice cream personal, and ice cream country). Among women, the SES was significantly negatively correlated with all portion measures except chicken personal portion size (see Table 4).

## 4. Discussion

To our knowledge, this study is the first to compare data on portion sizes from the USA, France, and a developing South American country. It uses a simple, picture-based size estimation technique that is easy to implement and produces consistent data. To our knowledge, it is the first study to probe perceived portion size norms and compare them to personal estimates.

The French, both men and women, report the smallest portion sizes across foods and for both personal and country portion sizes. It is notable that the French country estimates are much closer to their own portion sizes, suggesting a more communal, country-wide agreement on the proper amount to eat.

There are large gender differences, such that reported personal portion sizes for three foods were 28% larger for men than women. Across all foods, women reported a significantly larger difference between personal portion size and country estimate. It is possible that women, on average, recognized that their personal portions must be averaged with much larger men’s portions for their country, whereas men did not appear to compensate for their being only half of the population. Surprisingly, reported portion sizes did not correlate significantly with either weight or BMI but generally had a weak albeit significant relationship with SES, particularly among women. Personal (and country) portion sizes line up very roughly with national obesity rates. The degree of obesity in 2016 (22), from lowest to highest incidence, was France (21.6%), Brazil (22.1%), and the United States (36.2%).

The portion size results are robust and in line with prior work reporting smaller portion sizes in France (e.g., [14]). The portion size difference reported here, considerably greater in the USA than in France, is smaller than the effects reported by Rozin et al. in their observational studies in restaurants and supermarkets in France and the USA [14]. Given the self-report in the present study, there is a higher error potential than in the actual observations in the prior work. Also, the prior measurements were on a full range of adults rather than just university students, as in the present study. Finally, the present measurements were made about ten years after the prior study measurements. The data we report on now were collected roughly a decade ago, and since then, there has been a global pandemic and a global economic crisis, which may have impacted people’s perceptions of appropriate portion sizes. The type of results we report (particularly the cultural differences) appear rather stable over time, but at a minimum our results give an estimate of some important cultural differences at the time of data collection. 

As we suggested in the introduction and as appears in several papers and reviews [40,41], a major determinant of how much people eat is how much they have been served. Actual portion size, as reported by people, is in substantial part a measure of how much they are served. In home-eating situations, an individual has a fair amount of control over portion size, but in restaurants and in buying individual portion foods (such as small yogurt containers or soda cans), the size is determined externally. Thus, in part, the smaller portion size we report is a manifestation of smaller serving sizes in France. Of course, the served portion is a constraint on how much is eaten but does not strictly control it. As mentioned in the introduction, although it is almost always possible (at some cost) to order and eat two portion sizes, because of “unit bias”, people are disinclined to take two portions, so long as the portions are of modest size [44]. On the other hand, although there is a tendency to eat all that is served, if it is palatable, and there are often social forces promoting this, it is possible to consume less than the amount served.

There is greater social structure and more ritual in eating in France (as opposed to the USA), and greater respect for French cuisine and allegiance to it, compared to America [46]. The fact that we find that the difference between French personal and country portion sizes is much smaller than for the other three countries speaks to greater links between individual behaviors and cultural standards. Another important cultural difference to consider in future research is the social pressure to finish all the food that one is served (or that one serves oneself), which past studies have shown to be stronger in Western than East Asian cultural contexts [48,49,50].

Our data confirm past research on gender differences in attitudes about food [46,47], showing that men chose larger portions for themselves and that women believe they should eat less than others in their country. Almiron-Roig et al. [22] affirmed that men showed greater errors in estimation than women, but the gender differences may reflect a biological response to the higher energy needs of men. Also, a more significant error by the men would not, by itself, account for the mean difference.

Our finding of non-significant correlations between reported portion sizes and BMI could be seen to question the validity of our portion size measure. However, reviews of the portion size literature do not cite consistent findings on the relation between body weight status and portion size [3,11]. As with previous work, we used BMI as a predictor of food consumption and, through correlational data, portion size. In finding no relation, we realized that body weight, not BMI, is the proper predictor of food intake and portion size. A person of low absolute weight but high BMI should not be expected to eat large portions. It is body weight that should be linked to energy expenditure and not BMI. As it turns out, we also find no significant correlation between body weight and our measure of portion size. 

We report small but significant negative correlations between socioeconomic status and personal portion size measures. The direction of our correlations is as predicted in that, in general, larger portions are cheaper per unit, which might be more important for poorer people. Also, at least in the USA, the pressure to be thin is stronger in the higher social classes, and obesity is more common in lower social classes in developed countries [51]. Colapinto et al. [52] suggested that income could be related to size-based preferences, but there is no direct evidence that people of a lower SES are more inclined to overserve from larger packages or overeat from larger portions than people of a higher SES. 

We are puzzled by two consistent findings having to do with country estimates. One is that they are reliably higher than personal estimates for all foods and countries. If subjects take their own estimates to be representative of their gender and know that men take larger portions than women, then women’s estimates of country norms should be higher than their personal estimates (which they are), and men’s country estimates should be lower than their personal estimates (which they are not), except for France. So, there appears to be some error in the way the men in our study are making their estimates. Methodologically, it would have been better if we asked respondents to estimate country values for their own gender.

We are also puzzled by the substantial correlations between personal and country portion sizes in virtually all cases. We are not sure how to explain this, but one possibility is a form of egocentrism, and the second is carelessness, which might cause people to put the same value on two successive questions.

The chief limitations of our study are that it is restricted to self-reporting and our sample consists only of university students. As such, we have no assurance that our university student samples are representative of their own university. Although the perceived portion size for self and country are of interest in and of themselves, for a determination of actual portion size, direct observations or measurements in experiments in at least moderately realistic settings are more desirable. On the other hand, the study’s large sample size provides high statistical power both to uncover differences in portion sizes and identify relationships between portion size and key demographic variables. We should also note that although we believe our portion size visual aids (deck of cards, 16 oz cup, and tennis ball) were intuitive and easy to use, we only explicitly specified the exact size of the cup and the exact dimensions of playing cards can vary slightly from country to country. As tennis is an international sport, the size of the tennis ball is specified (diameter between 2.57 and 2.70 in) and is the same across countries [53]. In some countries, ice cream is commonly served in hemispherical half-scoops, so researchers should consider whether using a full tennis ball or one cut in half would be more appropriate. 

Portion size and, more generally, availability issues are very important components of food intake to be ignored. This is especially true because they are rather easy to manipulate in both experimental and real-world settings. American soda producers have recently introduced smaller cans than the prior 12-ounce American standard. It is possible to maintain profits while selling smaller portions, and an analysis of over a decade of sales data of soda in the USA shows that smaller package sizes performed better [54]. That said, consumers tend to respond negatively to the practice of “shrinkflation”, or reducing the size of a product without also reducing the price [55,56,57], particularly as inflation continues to rise in many parts of the world.

With respect to obesity, the critical issue is whether maintaining (over months or years) reduced portion sizes actually leads to a reduction in intake and weight. The long-term studies on this point have not yet been performed. The French-American data and a few lab studies that intervened for more than a week (referred to above) suggest that portion control can be a significant component of weight reduction. There is also some evidence that reducing portion size or plate size can reduce the amount of food that people waste, although more work is needed to clarify how changing portion sizes can minimize waste [58]. 

More work is needed in many countries on how actual portion sizes work with different foods and in other contexts. Benton [1] (p. 988) has argued that the effect of portion size on energy intake needs more study because there are many confounding variables, and “if the approach is to make a practical contribution, methods of changing portion sizes will need to be developed. However, this may prove to be a problem in a free market given that value for money is an important motivator”. 

## 5. Conclusions

Our results confirm past research on French-American differences in portion size by using a new visual tool. Our results indicate clearly that both personal and estimated country portion sizes are the smallest in France. We also show that French estimates of country portion sizes are much closer to their personal size, which could be related to stronger cultural norms around appropriate eating behavior in France. We also confirm past research on gender differences in portion sizes, such that men report larger portion sizes, and introduce new data from Brazil, which can help understand the recent changes in the eating culture and rates of obesity in these countries. 

In future research, portion size choices could be evaluated in multiple settings with different measures (e.g., a standardized visual array vs. estimated grams), and examining which of these measures best corresponds to actual intake. Furthermore, more cross-cultural work is needed to better understand the impact of portion size control (e.g., varying package and suggested serving sizes) on actual eating behavior. 

## Figures and Tables

**Table 1:** Demographic data of the samples of university students from three countries (*N* = 1695).

Country	Gender	*n*	Age in Years *M* (*SD*)	Body Mass Index *M* (*SD*)	SES *M* (*SD*)
Brazil	Women	360	21.3 (2.41)	22.5 (3.37)	3.1 (0.80)
	Men	223	21.2 (2.55)	22.3 (3.48)	3.1 (0.82)
France	Women	274	21.5 (1.41)	21.0 (2.24)	3.8 (0.83)
	Men	167	21.7 (1.53)	22.4 (2.28)	3.8 (0.99)
USA	Women	238	21.8 (3.27)	22.0 (3.29)	3.7 (0.96)
	Men	129	20.9 (3.04)	23.2 (2.74)	3.4 (0.93)

Note. SES = socioeconomic status (1 = lower, 2 = lower middle, 3 = middle, 4 = upper middle, and 5 = upper).

**Table 2:** Personal and country portion sizes for university students from three countries (*N* = 1695).

Country	Gender	Chicken Personal *M* (*SD*)	Chicken Country *M* (*SD*)	Soda Personal *M* (*SD*)	Soda Country *M* (*SD*)	Ice Cream Personal *M* (*SD*)	Ice Cream Country *M* (*SD*)
Brazil	Women	3.10 (1.18)	4.68 (1.26)	5.13 (1.73)	6.91 (1.13)	5.78 (2.19)	6.49 (1.92)
	Men	4.27 (1.71)	4.65 (1.19)	6.25 (1.75)	6.53 (1.42)	6.30 (2.32)	6.16 (2.00)
France	Women	2.58 (1.10)	3.24 (1.12)	4.24 (1.71)	5.38 (1.38)	4.35 (1.50)	4.76 (1.37)
	Men	4.01 (1.60)	3.49 (1.31)	5.23 (1.85)	4.98 (1.50)	5.25 (2.12)	4.94 (1.42)
USA	Women	3.19 (1.25)	4.92 (1.33)	4.48 (1.94)	6.93 (1.22)	5.22 (2.02)	7.01 (1.82)
	Men	4.64 (1.44)	5.47 (1.41)	5.52 (1.92)	6.80 (1.14)	5.85 (2.21)	7.00 (1.77)

Note. Ice cream scores were divided by 10 to put them on a similar metric to the other foods.

**Table 3:** Gender by country ANOVAs for personal portion size and country norm (*N* = 1695).

Variable	*F*(1) Gender	*F*(2) Country	*F*(2) Gender × Country	Significant Country Differences
Personal Portion Size				
Chicken	311.10 ***	20.93 ***	1.61	USA and BR > FR
Soda	105.86 ***	26.24 ***	0.14	BR > FR, USA
Ice Cream	30.44 ***	44.20 ***	1.28	BR > USA > FR
Country Portion Size				
Chicken	12.81 ***	220.19 ***	5.46 **	USA > BR > FR
Soda	17.41 ***	216.73 ***	1.22	USA and BR > FR
Ice Cream	0.28	153.64 ***	2.58	USA > BR > FR
Country minus Personal				
Chicken	186.08 ***	77.71 ***	1.26	USA > BR > FR
Soda	187.62 ***	60.14 ***	0.85	USA > BR > FR
Ice Cream	69.87 ***	47.93 ***	1.29	USA > BR and FR

Note: ** *p* < 0.01, *** *p* < 0.001. Pairwise comparisons used Scheffe’s test. BR = Brazil, FR = France, and USA = United States of America.

**Table 4:** Correlations between portion sizes, BMI, weight, and SES (*N* = 1695).

	1	2	3	4	5	6	7	8	9
1. Chicken Personal	-	**0.47**	**0.18**	**0.10**	**0.12**	**0.11**	0.06	0.01	−0.02
2. Chicken Country	**0.41**	-	**0.11**	**0.36**	**0.11**	**0.34**	0.07	0.06	**−0.13**
3. Soda Personal	**0.14**	0.08	-	**0.41**	**0.21**	0.07	0.08	0.05	**−0.14**
4. Soda Country	0.08	**0.32**	**0.39**	-	**0.18**	**0.38**	0.07	0.06	**−0.19**
5. Ice Cream Personal	**0.24**	**0.12**	**0.21**	**0.13**	-	**0.64**	0.07	0.06	**−0.21**
6. Ice Cream Country	0.10	**0.38**	0.05	**0.38**	**0.55**	-	0.06	0.05	**−0.16**
7. BMI	0.01	−0.04	−0.01	0.00	−0.01	0.02	-	**0.83**	**−0.10**
8. Weight	0.05	−0.09	−0.08	−0.08	−0.03	−0.03	**0.82**	-	−0.08
9. SES	0.02	−0.04	−0.09	**−0.12**	**−0.18**	**−0.11**	−0.01	**0.15**	-

Note. BMI = body mass index. SES = socioeconomic status. Correlations for men (*n* = 511–519) are in the lower left, and for women (*n* = 861–872) in the upper right. Correlations significant at *p* < 0.01 are in bold.

## Data Availability

A copy of the anonymized dataset is available at https://osf.io/umer3/?view_only=8280bfd1bfc142de95a967a6326ab779, accessed on 1 December 2023.
